# *In vitro* endothelial cell migration from limbal edge-modified Quarter-DMEK grafts

**DOI:** 10.1371/journal.pone.0225462

**Published:** 2019-11-20

**Authors:** Alina Miron, Daniele Spinozzi, Sorcha Ní Dhubhghaill, Jessica T. Lie, Silke Oellerich, Gerrit R. J. Melles

**Affiliations:** 1 Netherlands Institute for Innovative Ocular Surgery, Rotterdam, The Netherlands; 2 Melles Cornea Clinic Rotterdam, Rotterdam, The Netherlands; 3 Amnitrans EyeBank Rotterdam, Rotterdam, The Netherlands; Stein Eye Institute, University of California, Los Angeles, UNITED STATES

## Abstract

Endothelial cell migration plays a crucial role in achieving corneal clearance after corneal transplantation when using smaller-sized endothelial grafts to increase the donor pool. In this study we investigated how different strategies of Quarter-Descemet Membrane Endothelial Keratoplasty (Quarter-DMEK) limbal graft edge modification influence peripheral endothelial cell migration in an *in vitro* culture environment. For this study, 15 Quarter-DMEK grafts, prepared from 7 corneas deemed ineligible for transplantation but with intact and viable endothelial cells, were embedded in a cooled biocompatible, thermoresponsive matrix for culture. The limbal edge of ten Quarter-DMEK grafts were modified, either by using a small diameter punch or by peripheral radial cuts. All Quarter-DMEK grafts showed substantial collective endothelial cell migration from the radial cut graft edges, as observed by light microscopy at standardized time intervals. Grafts were retrieved from the polymer matrix after the two-week culture for immunohistochemistry analyses of the newly formed cell monolayers; this demonstrated the presence of tightly packed and viable cells that showed higher migratory ability at the leading edge. Peripheral endothelial cell migration, however, was not triggered by increasing cell exposure to free space through surgical modifications of the far periphery. Our data suggest that alterations in the far peripheral area of Quarter-DMEK grafts were insufficient to triggering cell migration from the limbal graft edge. This may be due to transient-amplifying cells that reside in the far periphery and which lack cytokinetic directional cues. Understanding the migration capacity of the peripheral endothelium could unlock cells’ therapeutic potential which are, at present, routinely discarded from transplantation. Encouraging peripheral cell migration may also improve clinical outcomes from Quarter-DMEK, but a more effective solution is required prior to clinical implementation of modified grafts.

## Introduction

We have recently introduced several modifications to Descemet membrane endothelial keratoplasty (DMEK), including Quarter-DMEK, as a means to potentially quadruple the availability of usable grafts from a single donor cornea [1–3]. The clinical outcomes are encouraging, use less tissue and the first series of Quarter-DMEK-eyes achieved visual outcomes comparable to conventional DMEK [1,2]. At the slit-lamp, however, Quarter-DMEK eyes typically showed a different corneal edema clearance pattern, due to the mismatch between the patient’s round descemetorhexis and the quadrant shape of the graft. This mismatch results in areas of bare stroma that must be cleared by migrating endothelial cells (EC). Clinically, clearing occurs most efficiently in the area adjacent to the radial cut graft edges, but tends to be slow and stagnant along the “limbal” round edge of the Quarter-DMEK-grafts.

We have replicated the cell migration of the Quarter-DMEK grafts in an *in vitro* culture system in our previous experiments [4]. The results confirmed that endothelial cell migration readily occurred along the radial cut edges of the grafts, but that migration from the peripheral limbal edge was not observed. This finding prompted us to question the role played by the peripheral endothelium as a cell source in the restoration of corneal clearance. We hypothesized that the arrangement of fibrillary bands of collagen in the periphery could act a as a barrier or “fence”, thereby preventing migration.

This study’s purpose was to evaluate whether peripheral edge modification could break down the physical barrier that inhibits cell migration from the limbal edge of the graft, thereby promoting cell migration in a manner similar to the radial edges.

## Materials and methods

### Corneas

Seven human postmortem corneas, which were ineligible for transplantation but which had an intact and viable endothelial cell layer (from five donors (mean age 69 (±4) years; range 61–73 years)) (**Table 1**), were obtained from Amnitrans EyeBank Rotterdam. All donors stated that they had no objection to transplant-related research and the study adhered to the tenets of the Declaration of Helsinki. No institutional review board approval was obtained, given that no approval is required for this kind of research if no extra procedure was performed to obtain the samples and if donors had consented to having the samples used for research purposes, according to a national regulation (https://www.ccmo.nl/onderzoekers/soorten-onderzoek/niet-wmo-onderzoek/onderzoek-met-lichaamsmateriaal).

**Table 1:** Donor demographics.

Donor Information	Indicators
Number of corneas/donors	7/5
Gender	
Female	2
Male	3
Mean age (±SD), yrs (range)	69 (±4), (61–73)
Mean storage time* (±SD), days (range)	6 (±3), (2–12)
Cause of death	
Cardio/Stroke	2
Respiratory	2
Malignant neoplasm	1

*Mean storage time = time between death and culture of isolated DM-EC tissue

SD = standard deviation

yrs = years

### Quarter-Descemet membrane endothelial keratoplasty graft preparation

The Quarter-DMEK grafts were prepared at Amnitrans EyeBank Rotterdam, as described previously [3], by a single eye bank technician (JTL) with extensive experience in DMEK and modified DMEK techniques [5]. For every cornea, two of the four Quarter-DMEK grafts were modified, by either using a 1 mm diameter biopsy punch (Kai Europe GmbH, Solingen, Germany) to create two cuts in the periphery or by using an ophthalmic keratome (MANI, INC. Tochigi, Japan) to create 3 peripheral radial cuts. One Quarter-DMEK graft was used as a backup in case of failed preparation of modified grafts, and one unmodified Quarter-DMEK graft was used as a negative control. A positive control was created by cutting the peripheral edge off from a Quarter-DMEK graft, thereby creating a triangle-shaped endothelial graft. The endothelial cell density (ECD) determined in the eye bank after DMEK graft preparation was on average 2,514 (±267) cells/mm^2^ for all seven corneas. Each Quarter-DMEK graft was then stored separately in organ culture medium (CorneaMax, Eurobio) for fewer than 24 hours before being evaluated for chemotactic cell ability.

### Preparation of Mebiol® Gel culture medium

Mebiol® Gel was obtained in a lyophilized and sterilized flask from Bio-Connect B.V. (Huissen, The Netherlands). The gel was reconstituted in 50 ml of culture medium, consisting of Dulbecco’s modified eagle medium (DMEM) supplemented with 15% fetal bovine serum (FBS), 2 mM L-Glutamine, 2 ng/ml fibroblast growth factor (bFGF), 0.3 mM L-ascorbic acid 2-phosphate (all from Sigma-Aldrich, Zwijndrecht, The Netherlands), and 10,000U-ml Pen/Strep (Carl Roth GmbH + Co. KG, Karlsruhe, Germany). The culture-flask was then refrigerated at 4°C for about three hours yielding a viscous transparent Mebiol® Gel-culture medium mixture. The uniform liquid sol was then carefully aliquoted, to avoid air bubbles, at desired volumes (1.5–4 ml) and was stored at -20°C for later use.

### Cell migration study

15 individual Quarter-DMEK rolls with the trabecular membrane (TM) attached, obtained from 7 different corneas, were unfolded endothelial-side-up on FNC-coated (fibronectin, collagen, and albumin coating mix, Athena ES^TM^ Baltimore, MD, USA) glass coverslip and evaluated *in vitro* in order to examine whether peripheral edge modifications of a Quarter-DMEK graft could trigger cell migration. Unfolding of all grafts was performed in a “minimal touch” manner by grabbing the graft only at the TM site with a McPherson forceps, and dropping organ-culture medium onto the graft while the glass coverslip was kept tilted at a small angle.

The TM was then carefully removed from the Quarter-DMEK grafts and the endothelium was submerged in serum-containing culture medium in order to ensure cell viability during the experiments. Each glass coverslip, which supported one Quarter-DMEK graft, was transferred to a 24-well plate and a drop of liquefied gel-culture medium mixture (temp 4–8°C) was placed at the center of the well. The gel became more solid when kept at 37°C for about 5 minutes. Once the gel became firmer, the well was then completely filled with cooler liquid drops of the gel-culture medium mixture (~500 μl end volume), thereby preventing Quarter-DMEK grafts gliding over the solid support. Subsequent incubation at 37°C for about 10 minutes led to a solidified gel matrix that was uniformly distributed over the Quarter-DMEK grafts. Growth factors and nutrients were provided by keeping the gel surface moist using 300 μl of culture medium. Grafts were cultured over 2 weeks in a humidified atmosphere at 37°C with 5% CO_2_. Medium was refreshed every 2–3 days. Quarter-DMEK grafts were photographed daily with an AxioVert.A1 microscope with AxioCam ERc 5s stand-alone functionality camera (Zeiss, Oberkochen, Germany) to examine cell morphology and the degree of cell migration.

The recovery of the Quarter-DMEK grafts after cultivation was performed by cooling the gel below the sol-gel transition temperature (<20 ^o^C). Firstly, the warm culture medium was replaced by cold fluid (PBS or DMEM) and gently aspirated 5 minutes later, removing the uppermost liquefied layer of the gel in the process. This was performed several times until the gel was removed completely, without disturbing the graft position in the well.

### Immunohistochemistry

Quarter-DMEK samples, reclaimed from gel culture for immunohistochemistry analysis, were first fixed in 4% paraformaldehyde (Sigma Aldrich, The Netherlands) for 15 minutes. Following fixation, the grafts were then washed with phosphate-buffered saline (PBS), permeabilized using permeabilization buffer (0.1% Triton X-100 in PBS, Sigma Aldrich, The Netherlands) and finally incubated with blocking buffer (5% bovine serum albumin in PBS, Sigma Aldrich, The Netherlands) for 30 minutes in order to prevent non-specific staining. Blocking buffer was also used for both primary and secondary antibody (Life Technology, The Netherlands) dilutions.

Samples were stained for the expression of zonula occludens-1 (ZO-1), vimentin and Na^+^/K^+^-ATPase to establish the baseline endothelial morphology and CD73 for potential cell migration. Incubation with primary antibodies was performed at the following dilutions; anti-ZO-1 tight junction protein (anti-ZO-1/TJP1, dilution 1:100), anti-vimentin filamentous protein (anti-vimentin, dilution 1:100), anti-sodium/potassium-ATPase (anti-Na^+^/K^+^-ATPase β1, dilution 1:100) and anti-lymphocyte differentiation antigen CD73 (anti-CD73, single purchase from Abcam, Cambridge, United Kingdom, dilution 1:100). Incubations were performed for 1 hour and were then followed by several PBS washing steps. Samples were then incubated with fluorescent secondary antibodies that had been conjugated to Alexa Fluor® (dilution 1:200) for 45 minutes. After washing with PBS, the samples were stained with 4',6-diamidino-2-phenylindole (DAPI, Sigma-Aldrich, The Netherlands) to visualize the nuclear DNA, and were then imaged using an inverted fluorescence microscope connected to a camera (Axiovert, Zeiss).

### Cell viability assay

The membrane-permeable dye Calcein-AM (Sigma-Aldrich Chemistry BV, Zwijndrecht, The Netherlands) was received as stock solution of 4 mM in dimethylsulfoxide at -20°C, prepared as a working solution of 400 μM in PBS and added directly to grafts in order to examine the cell viability of Quarter-DMEK grafts after the culture period. A 45-minute incubation period at room temperature allowed for the nonfluorescent Calcein-AM to be hydrolyzed by intercellular esterases into the highly negatively charged green fluorescent Calcein, which is retained in the cell cytoplasm. After one more PBS washing step, grafts were ready for imaging by microscopy.

## Results

### Cell migration study

**Fig 1:**
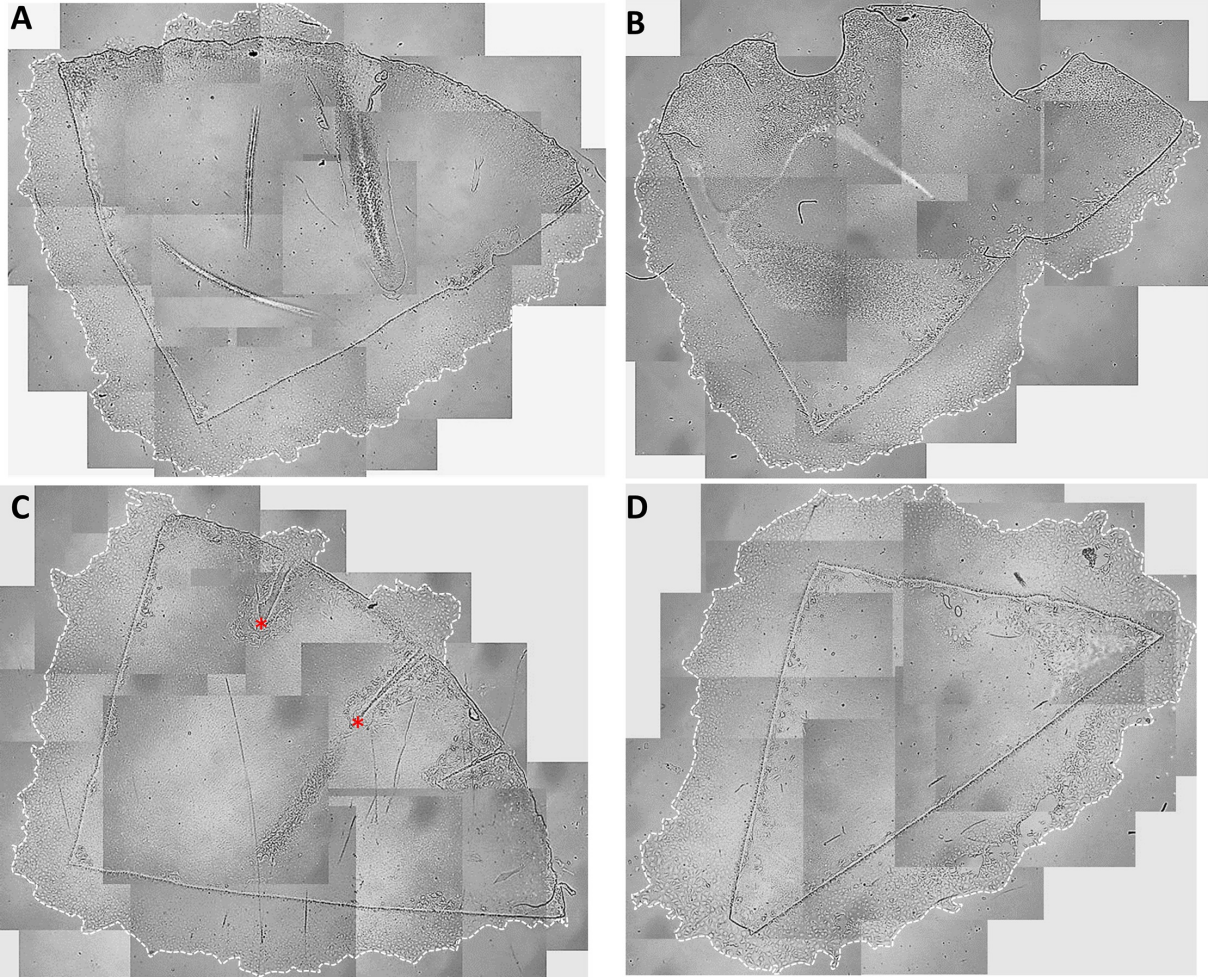
Representative images of each type of Quarter-DMEK graft modification and Quarter-DMEK graft controls after two weeks *in vitro* gel culture. Collage of light microscopy images (x25 magnification) to create an overview of the (A) Quarter-DMEK graft with intact far periphery showing lack of cell migration along the limbal round edge (negative control), (B) fence-broken Quarter-DMEK graft for which the periphery was punched with a 1 mm diameter biopsy punch twice, showing no stimulation of cell migration, (C) Quarter-DMEK graft for which the periphery was radially cut three times with an ophthalmic knife showing cell migration initiated only from those deep cuts (red marks) bypassing the intermingled fibrillary area and opening the endothelium’s periphery, (D) Quarter-DMEK graft with a cut off periphery showing uniform cell migration from all cut edge sites (positive control). The dashed line outlines the cell monolayer migration edge.

All grafts placed in culture showed cell migration along the radial cut edges up to the tip of the graft (**Fig 1**). The degree of migration decreased towards the peripheral graft area in both the limbal edge-modified and unmodified Quarter-DMEK grafts (negative control) in which the dense fibrillary area was exposed to the open space (**Fig 1A–1C**). The peripheral edge modification of the graft in order to cut through the dense fibrillary area either with the biopsy punch or radial cuts, did not sufficiently stimulate cell migration to populate the limbal round edge of the graft (**Fig 1B and 1C**). Greater cell migration was seen when the cuts were very deep and protruding into the paracentral zone of the endothelium (**Fig 1C**). The positive control graft, where the fibrillary area was completely removed, showed a uniform cell migration pattern all around the cut edges (**Fig 1D**).

**Fig 2:**
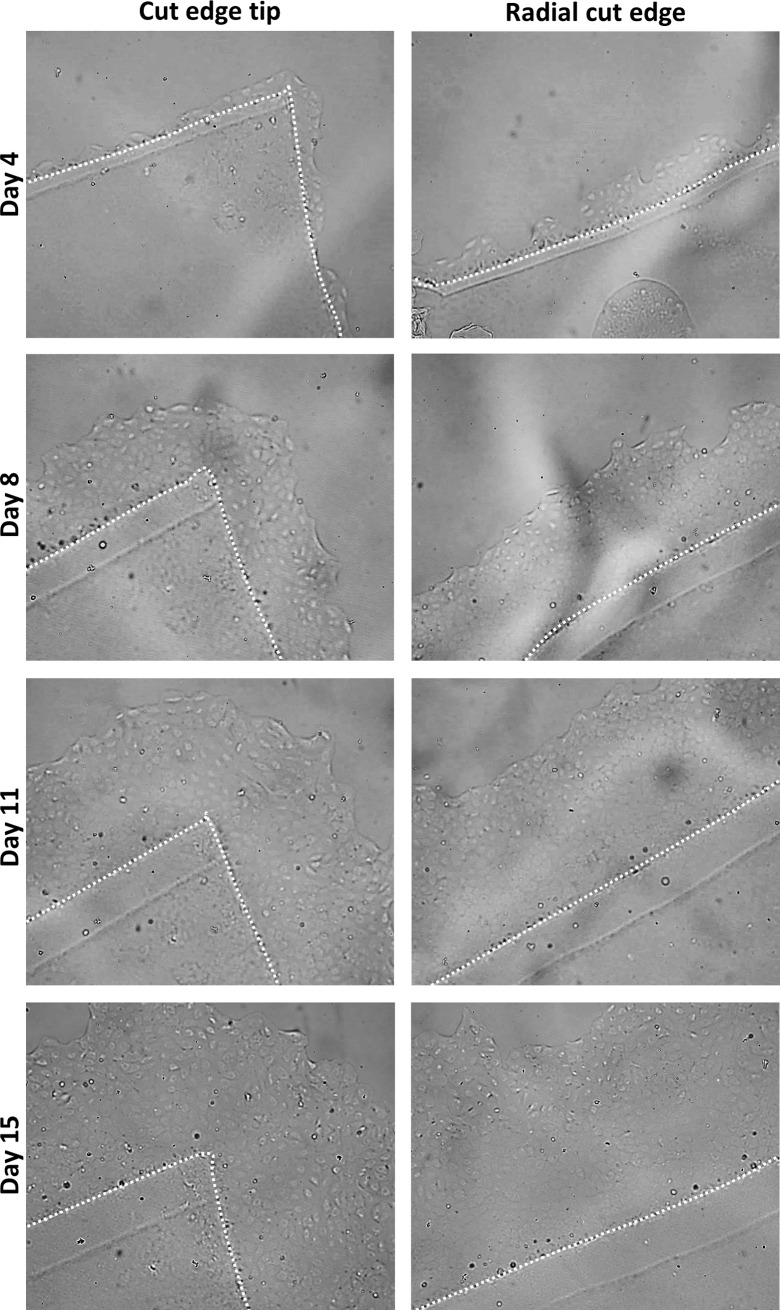
Example of collective *in vitro* endothelial cell migration from gel-cultured Quarter-DMEK grafts. Light microscopy images of the cut edge tip (Left) and the radial cut edge (Right) of a Quarter-DMEK graft taken at Day 4, Day 8, Day 11, and Day 15 (Top to Bottom view) with 100x magnification. Around the cut edge tip and along the radial cut edges of the Quarter-DMEK graft collective endothelial cell migration in a form of a monolayer was observed; leader cells at the front edge of the advancing cell sheet are identifiable; collective migration pattern was most evident at Day 8. The latter appear out of focus and hazy due to the difference in focal plane between the migrated cells and the cells on the graft itself (height difference due to Descemet membrane). In each of the photos, the dashed line outlines the edge of the Quarter-DMEK graft. The double-lined cut edge of the graft in all images is an optical aberration caused by the gel matrix.

All grafts showed substantial cell migration from day 4 up to day 15 (**Fig 2**) which was when the graft was retrieved for immunohistochemistry evaluation. Cells migrated in interconnected groups (i.e. collective cell migration) with a “leading” cell group seen at the front edge in all grafts (15/15).

### Immunohistochemistry

**Fig 3:**
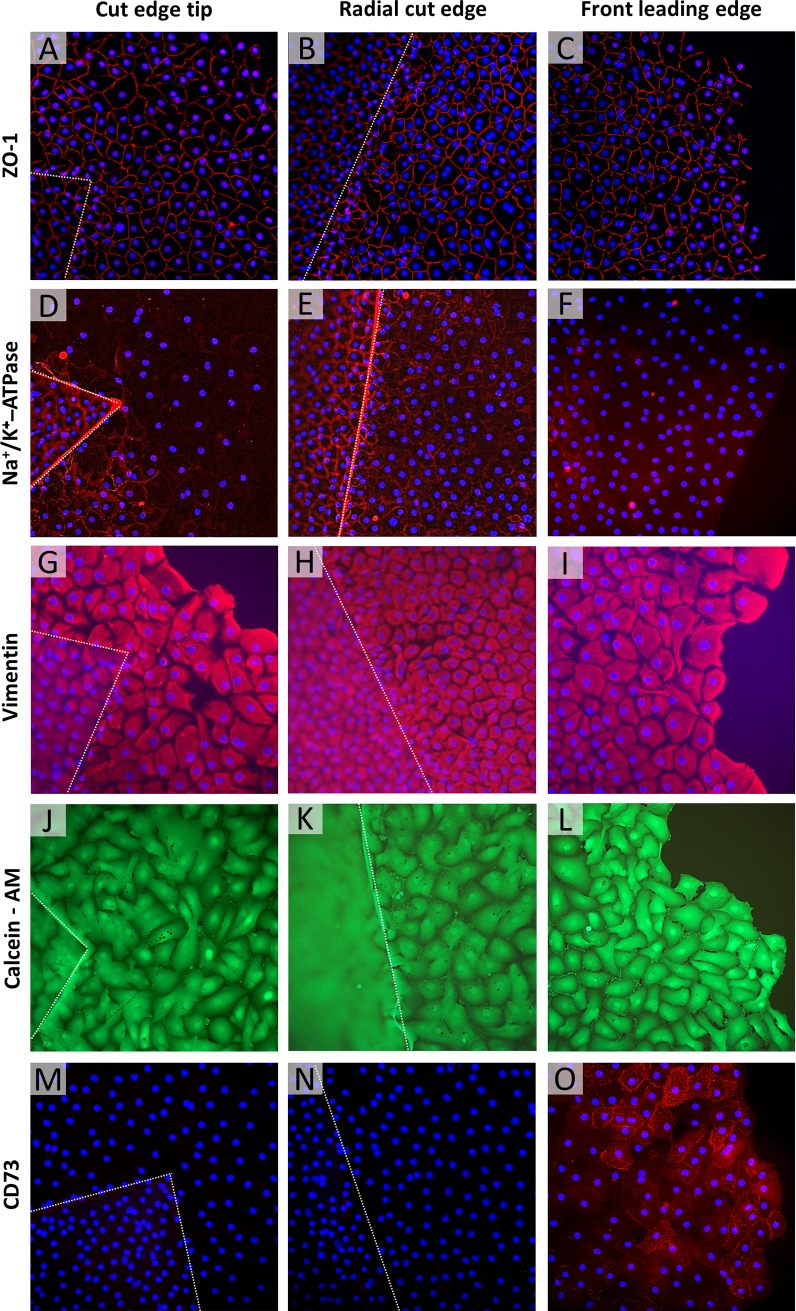
Immunofluorescence staining of the newly formed cell monolayer. Expression of ZO-1 (A-C), Na^+^/K^+^-ATPase (D-F) vimentin (G-I), Calcein-AM (J-L) and CD73 (M-O) was analyzed around the cut edge tip (Left), along the radial cut edge (Middle) of the Quarter-DMEK graft, and at the monolayer’s front leading edge contour (Right) after 17(±3) days in gel culture and subsequent gel removal. Characteristic expressions for the tight junction protein ZO-1 (A, B red) and functional protein Na^+^/K^+^-ATPase (D, E red) were uniformly observed along lateral cell borders across the monolayer (A, B red); however, cells at the front leading edge of the monolayer showed a discontinuous distribution of ZO-1 (C, red) and Na^+^/K^+^-ATPase (F, red). Strong expression of vimentin intermediate filaments was detected throughout the monolayer (G, H, I red) and in cells at the font leading edge more specifically, which demonstrate increased motility (I red). Cell viability evaluated by expression of Calcein-AM showed strong fluorescence intensity in the confluence monolayer of cultured cells (J, K, L green). CD73 expression marker was uniquely associated with cells near the leading edge (O red) indicating involvement in the process of cell migration; a lack of CD73 expression in the confluent monolayer indicates poor ability for cell migration and growth at the rear of the leading edge (M, N red). Due to the difference in focal plane between the migrated cells and the cells on the graft itself (height difference due to Descemet membrane), the latter appear out of focus and hazy. In each of the photos, the dashed line outlines the edge of the Quarter-DMEK graft, 200x magnification.

**Fig 4:**
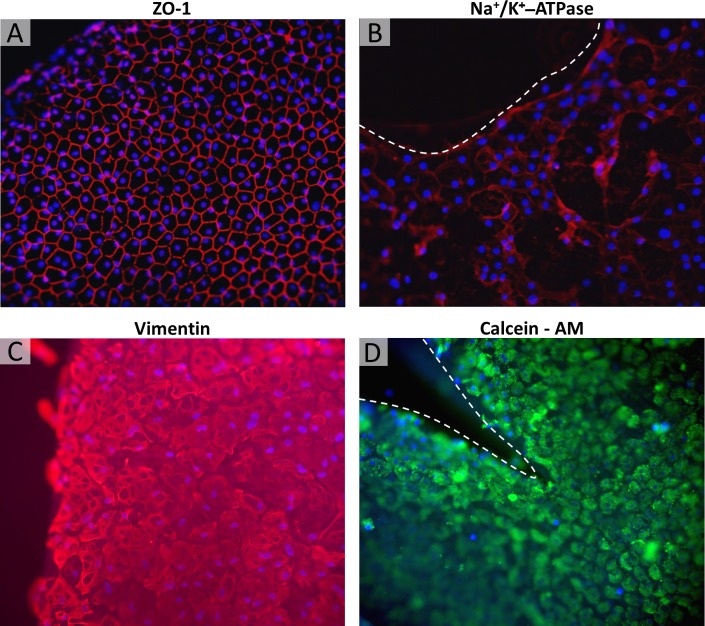
Immunofluorescence staining of the Quarter-DMEK graft with focus on far periphery. Expressions for the tight junction protein ZO-1 (A: control graft, red), functional protein Na^+^/K^+^-ATPase (B: graft with trephined periphery, red), and structural protein vimentin (C: control graft, red) counterstained with DAPI (blue) showed sparsely distributed cells with irregular morphology and altered pump function activities in the far periphery. The round limbal edge the Quarter-DMEK graft was populated by viable cells as indicated by Calcein-AM expressions (D: graft with radially cut-through periphery, green). Grafts were imaged after 17(±3) days in gel culture and subsequent gel removal 200x magnification.

Immunohistochemistry analysis confirmed the formation of a continuous functional monolayer created by direct cell migration into the free space available, apart from at the round edge of the graft (**Figs 3 and 4**). Typical expression profile was found for endothelial marker ZO-1 and Na^+^/K^+^-ATPase (**Fig 3A, 3B, 3D and 3E red**) in EC that depart from the radial graft cut edge up to the rear of the leading edge. Resistance barrier and the pump function protein showed a discontinuous expression over the front leading cell edge, the (**Fig 3C and 3F, red**) which is concomitant with strong polymerization of vimentin intermediate filaments (**Fig 3G, 3H and 3I, red**) and the expression of regulatory molecule CD73 which is involved in cell migration (**Fig 3M, 3N and 3O, red**). Cell viability, evaluated by expression of Calcein-AM, showed strong fluorescence intensity in the confluence monolayer of cultured cells (**Fig 3J, 3K and 3L green**).

Although migration was not observed over the round, limbal edge of the Quarter-DMEK graft, the Calcein-AM expression (**Fig 4D, green**) confirmed the presence of viable endothelial cells. Expressions for the tight junction protein ZO-1 (**Fig 4A, red**), functional protein Na^+^/K^+^-ATPase (**Fig 4B, red**), and structural protein vimentin (**Fig 4C, red**) showed sparsely distributed cells with irregular morphology and altered pump function activities.

## Discussion

We know, both from clinical experience [1–3] and from our previous *in vitro* work [4], that EC from the cut edges of a quarter DMEK graft have the capacity to migrate and spread throughout regions of bare stroma in recipient eyes. However, poor corneal clearance along the round graft edge [2] suggested that EC migration was almost absent in the far periphery, which was also confirmed *in vitro* [4]. Previously, He et al. suggested that the furrow-like distribution of the underlying collagen fibers in the corneal far periphery directs the migration of EC towards the center of the cornea throughout a person’s life, thereby limiting migration in the other direction [6]. We suspected that the lack of migration from the periphery was, therefore, due to this obstructive barrier, or “fence” and that, by breaking through it, cells with migratory potential could be unlocked.

Our results showed that EC migrated collectively from all Quarter-DMEK grafts’ radial cut edges. As they migrated through the gel matrix, the cells first displayed “mesenchymal collective migration” where individual satellite cells separate out from the group, elongate and independently stretch and branch into the free space. After a few days, this pattern changed to one of “direct protrusion formation”, that is that an area in which a sheet of cells coalesces behind the leading edge, and march together [7]. The leading edge protrusions were dynamic vimentin-containing structures with plasma membrane blebs polarized in the direction of the open space and regulated by activation of CD73 [8,9]. Interestingly, at the leading edge of migrating cells, Na^+^/K^+^ -ATPase pumps and ZO-1 expression were more irregular, suggesting that these leader ECs were reducing their pump function specialization in favor of migration, while the followers retain more of their EC properties [10,11]. Thus, the rear end of EC maintained cell-cell adhesions and displayed homogenous endothelial cell expression for structural (ZO-1) and functional (Na^+^/K^+^ -ATPase) markers.

Attempts to break the barrier by trephination or radial cuts, however, did not result in cell migration from the limbal round edge of a Quarter-DMEK graft within the study period of 2.5 weeks regardless the peripheral edge modifications. This suggests that the lack of migration from the periphery is due to a process that is more complex than a simple collagen barrier. It was also not due to the absence of viable of cells, since immunolocalization showed cells with expression of the structural (ZO-1 and vimentin) and functional markers (Na^+^/K^+^ -ATPase). The morphology was also different in this area with fewer cells displaying the typical endothelial hexagonal shape. This suggests that the cell-cell and cell-extracellular matrix (ECM) interactions are different to the central cornea and may prohibit migration. A stimulus greater than eliminating contact inhibition seems to be required in order to prompt these cells to move. It is possible that either the integrin-dependent adhesion between cells and underlining ECM is less expressed, thereby affecting cell tension and morphology, or that the level of GTPases, responsible for cell contractility and protrusion mediation at the leading edge, is reduced [12].

Clinically, the failure of far peripheral EC to migrate, in spite of modification, remains a limitation of the current quarter DMEK approach. However, cells located beneath the Schwalbe’s line have been found to have progenitor cell-like properties [13] and might play a critical role in the stabilization of the endothelial cell density after transplantation. Thus, understanding the nature of these peripheral endothelial cells, how they differ from the central cells, and how to encourage them to migrate would greatly improve the pool of donor tissue available for patients in need.

## Supporting information

S1 Fig Additional light microscopy and immunohistochemistry images of flattened grafts with and without modification of the limbal graft edge. Collage of light microscopy images (x25 magnification) to create an overview of (A) a Quarter-DMEK graft with intact far periphery and (D) a Quarter-DMEK graft with modification of the limbal graft edge; both after 2 weeks of *in vitro* gel culture. Both grafts show extensive cell migration along the radial cut graft edges as outlined by the dotted white line, but not along the limbal graft edge. (B, C) Cell viability evaluated by expression of Calcein-AM showed strong fluorescence intensity in the confluent monolayer of cultured cells at different positions of the migrated cell layer; Images shown in (B) and (C) correspond to the areas marked with one and two white asterisks in image (A), respectively. (E, F) Characteristic expression for the functional protein marker Na^+^/K^+^-ATPase observed across the monolayer. Images shown in (E) and (F) correspond to the areas marked with one and two red asterisks in image (D), respectively. (F) Cells close to the modified limbal edge showed discontinuous expression of Na^+^/K^+^-ATPase corroborating the finding that endothelial cells along the limbal edge differ morphologically from endothelial cells in the corneal center.
(PDF)
