# Supplementary material for: In vitro endothelial cell migration from limbal edge-modified Quarter-DMEK grafts
Source: PLoS One. 2019 Nov 20;14(11):e0225462. doi: 10.1371/journal.pone.0225462 (PMC6867638; doi:10.1371/journal.pone.0225462)
Supplement: S1 Fig — Collage of light microscopy images (x25 magnification) to create an overview of (A) a Quarter-DMEK graft with intact far periphery and (D) a Quarter-DMEK graft with modification of the limbal graft edge; both after 2 weeks of in vitro gel culture. Both grafts show extensive cell migration along the radial cut graft edges as outlined by the dotted white line, but not along the limbal graft edge. (B, C) Cell viability evaluated by expression of Calcein-AM showed strong fluorescence intensity in the confluent monolayer of cultured cells at different positions of the migrated cell layer; Images shown in (B) and (C) correspond to the areas marked with one and two white asterisks in image (A), respectively. (E, F) Characteristic expression for the functional protein marker Na+/K+-ATPase observed across the monolayer. Images shown in (E) and (F) correspond to the areas marked with one and two red asterisks in image (D), respectively. (F) Cells close to the modified limbal edge showed discontinuous expression of Na+/K+-ATPase corroborating the finding that endothelial cells along the limbal edge differ morphologically from endothelial cells in the corneal center. (PDF) [file pone.0225462.s001.pdf]

Supporting information

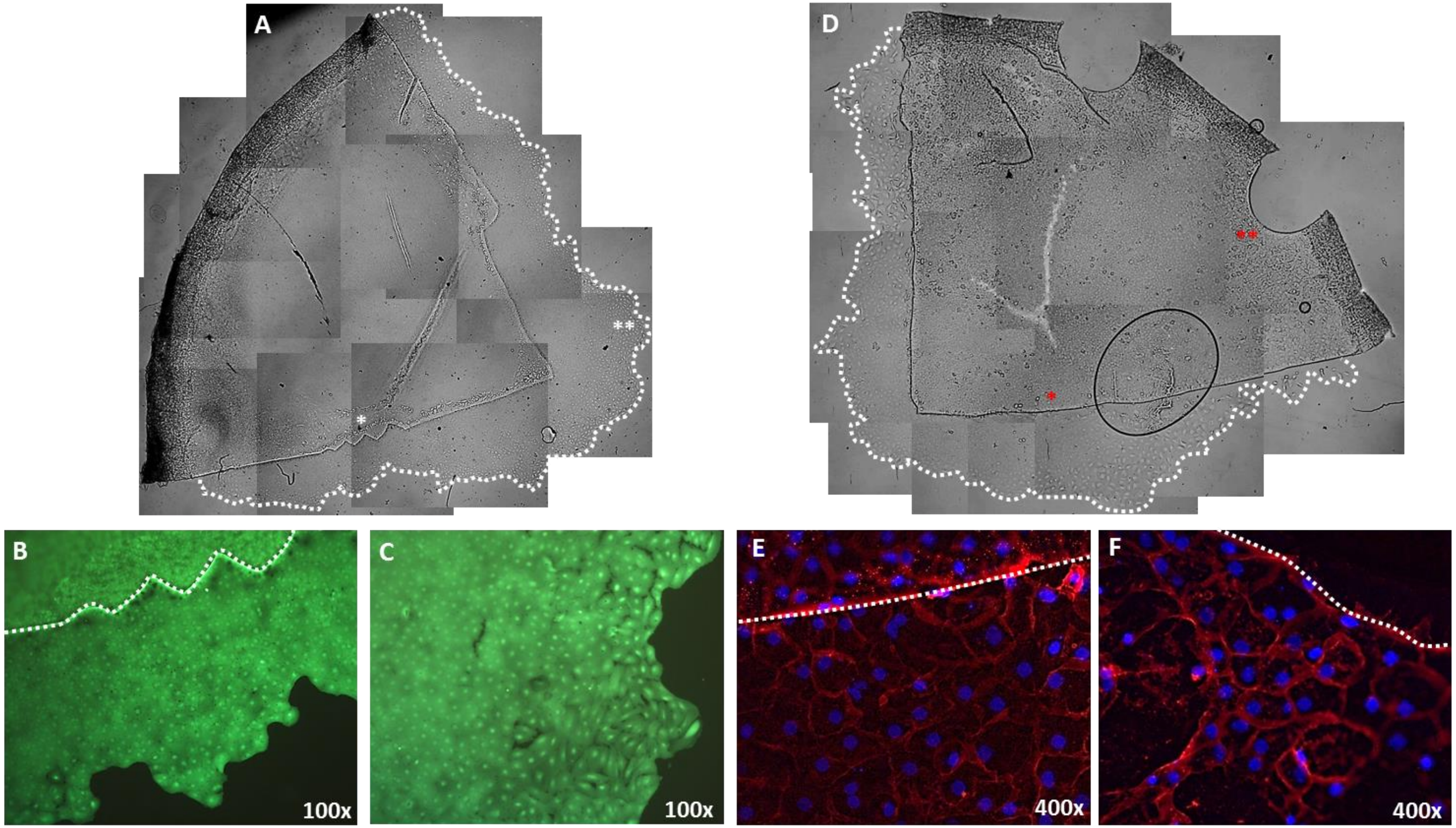

S1 Fig. Additional light microscopy and immunohistochemistry images of flattened grafts with and without modification of the limbal graft edge. Collage of light microscopy images (x25 magnification) to create an overview of (A) a Quarter-DMEK graft with intact far periphery and (D) a Quarter-DMEK graft with modification of the limbal graft edge; both after 2 weeks of in vitro gel culture. Both grafts show extensive cell migration along the radial cut graft edges as outlined by the dotted white line, but not along the limbal graft edge. (B, C) Cell viability evaluated by expression of Calcein-AM showed strong fluorescence intensity in the confluent monolayer of cultured cells at different positions of the migrated cell layer; Images shown in (B) and (C) correspond to the areas marked with one and two white

asterisks in image (A), respectively. (E, F) Characteristic expression for the functional protein marker Na^+/K^+-ATPase observed across the monolayer. Images shown in (E) and (F) correspond to the areas marked with one and two red asterisks in image (D), respectively. (F) Cells close to the modified limbal edge showed discontinuous expression of Na^+/K^+-ATPase corroborating the finding that endothelial cells along the limbal edge differ morphologically from endothelial cells in the corneal center.
